# Assessment of Outcomes From 1-Year Surveillance After Detection of Early Gastric Cancer Among Patients at High Risk in Japan

**DOI:** 10.1001/jamanetworkopen.2022.27667

**Published:** 2022-08-19

**Authors:** Yoshinobu Yamamoto, Naohiro Yoshida, Tomonori Yano, Takahiro Horimatsu, Noriya Uedo, Noboru Kawata, Hiromitsu Kanzaki, Shinichiro Hori, Kenshi Yao, Seiichiro Abe, Chikatoshi Katada, Chizu Yokoi, Ken Ohata, Hisashi Doyama, Kenichi Yoshimura, Hideki Ishikawa, Manabu Muto

**Affiliations:** 1 Department of Gastrointestinal Oncology, Hyogo Cancer Center, Akashi, Hyogo, Japan; 2 Department of Gastroenterology, Ishikawa Prefectural Central Hospital, Kanazawa, Ishikawa, Japan; 3 Department of Gastroenterology and Endoscopy, National Cancer Center Hospital East, Kashiwa, Chiba, Japan; 4 Department of Therapeutic Oncology, Kyoto University Graduate School of Medicine, Kyoto, Kyoto, Japan; 5 Department of Gastrointestinal Oncology, Osaka International Cancer Institute, Osaka, Osaka, Japan; 6 Division of Endoscopy, Shizuoka Cancer Center, Suntogun, Shizuoka, Japan; 7 Department of Gastroenterology and Hepatology, Okayama University Graduate School of Medicine, Dentistry and Pharmaceutical Sciences, Okayama, Okayama, Japan; 8 Department of Endoscopy, National Hospital Organization Shikoku Cancer Center, Matsuyama, Ehime, Japan; 9 Department of Endoscopy, Fukuoka University Chikushi Hospital, Chikushino, Fukuoka, Japan; 10 Endoscopy Division, National Cancer Center Hospital, Chuo, Tokyo, Japan; 11 Department of Gastroenterology, Kitasato University School of Medicine, Sagamihara, Kanagawa, Japan; 12 Endoscopy Division, National Center for Global Health and Medicine, Shinjuku, Tokyo, Japan; 13 Department of Gastroenterology, NTT Medical Center Tokyo, Shinagawa, Tokyo, Japan; 14 Department of Biostatistics, Hiroshima University Hospital, Hiroshima University, Hiroshima, Japan; 15 Department of Molecular-Targeting Cancer Prevention, Kyoto Prefectural University of Medicine, Kyoto, Japan

## Abstract

**Importance:**

Single endoscopic examination often misses early gastric cancer (GC), even when both high-definition white light imaging and narrow-band imaging are used. It is unknown whether new GC can be detected approximately 1 year after intensive index endoscopic examination.

**Objective:**

To examine whether new GC can be detected approximately 1 year after intensive index endoscopic examination using both white light and narrow-band imaging.

**Design, Setting, and Participants:**

This case-control study was a preplanned secondary analysis of a randomized clinical trial involving 4523 patients with a high risk of GC who were enrolled between October 1, 2014, and September 22, 2017. Data were analyzed from December 26, 2019, to April 21, 2021. Participants in the clinical trial received index endoscopy to detect early GC via 2 examinations of the entire stomach using white light and narrow-band imaging. The duration of follow-up was 15 months. The secondary analysis included 107 patients with newly detected GC (case group) and 107 matched patients without newly detected GC (control group) within 15 months after index endoscopy.

**Interventions:**

Surveillance endoscopy was scheduled between 9 and 15 months after index endoscopy. If new lesions suspected of being early GC were detected during surveillance endoscopy, biopsies were obtained to confirm the presence of cancer.

**Main Outcomes and Measures:**

The primary end point was the rate of new GC detected within 15 months after index endoscopy. The main secondary end point was identification of risk factors associated with new GC detected within 15 months after index endoscopy.

**Results:**

Among 4523 patients (mean [SD] age, 70.6 [7.5] years; 3527 men [78.0%]; all of Japanese ethnicity) enrolled in the clinical trial, 4472 received index endoscopy; the rate of early GC detected on index endoscopy was 3.0% (133 patients). Surveillance endoscopy was performed in 4146 of 4472 patients (92.7%) who received an index endoscopy; the rate of new GC detected within 15 months after index endoscopy was 2.6% (107 patients). Among 133 patients for whom early GC was detected during index endoscopy, 110 patients (82.7%) received surveillance endoscopy within 15 months after index endoscopy; the rate of newly detected GC was 10.9% (12 patients). For the secondary analysis of risk factors associated with newly detected GC, characteristics were well balanced between the 107 patients included in the case group vs the 107 patients included in the matched control group (mean [SD] age, 71.7 [7.2] years vs 71.8 [7.0] years; 94 men [87.9%] in each group; 82 patients [76.6%] vs 87 patients [81.3%] with a history of gastric neoplasm). Multivariate analysis revealed that the presence of open-type atrophic gastritis (odds ratio, 6.00; 95% CI, 2.25-16.01; *P* < .001) and early GC detection by index endoscopy (odds ratio, 4.67; 95% CI, 1.08-20.21; *P* = .04) were independent risk factors associated with new GC detection.

**Conclusions and Relevance:**

In this study, the rate of new GC detected by surveillance endoscopy approximately 1 year after index endoscopy was similar to that of early GC detected by index endoscopy. These findings suggest that 1-year surveillance is warranted for patients at high risk of GC.

## Key Points

**Question:**

Is new gastric cancer (GC) detectable approximately 1 year after intensive index endoscopic examination using both white light and narrow-band imaging among patients with a high risk of GC?

**Findings:**

In this case-control study involving secondary analysis of a randomized clinical trial of 4523 patients with a high risk of GC, the rate of new GC detected within approximately 1 year after intensive index endoscopic examination was 2.6%, which was similar to the rate of early GC detected by index endoscopy (3.0%).

**Meaning:**

This study’s findings suggest that 1-year surveillance is warranted among patients with a high risk of GC even after intensive endoscopic examination using both white light and narrow-band imaging.

## Teaser

This case-control study involving a secondary analysis of a randomized clinical trial assesses whether new gastric cancer can be detected approximately 1 year after early gastric cancer detected by intensive index endoscopic examination among patients with a high risk of gastric cancer.

## Introduction

Gastric cancer (GC) has the fifth highest incidence and the fourth highest mortality rate worldwide, with an estimated 1 089 103 new cases and 768 793 deaths in 2020.^1^ The 5-year survival rate is less than 10% when patients are diagnosed at an advanced stage.^1^ However, if patients with GC are diagnosed at an early stage (eg, stage I), their prognosis is good. A prospective confirmatory study by the Japan Clinical Oncology Group (JCOG0607)^2^ examining curative endoscopic submucosal dissection for clinically mucosal GC revealed an excellent 5-year survival rate of 97.0%. In addition, endoscopic resection of mucosal GC can preserve the organ. Hence, detection of early GC is important to improve prognosis and preserve the organ.^3^

First-generation narrow-band imaging was useful to detect early cancer in the esophagus and head and neck region^4^; however, this imaging was not suitable for observation of the stomach due to insufficient light intensity. To overcome this disadvantage, second-generation narrow-band imaging increased the light intensity and improved the resolution. To investigate the usefulness of second-generation narrow-band imaging for the detection of early GC, a multicenter prospective randomized clinical trial was conducted,^5^ which reported that the real-time detection rate of early GC by second-generation narrow-band imaging was not superior to that of conventional white light imaging in patients with a high risk of GC. In this clinical trial,^5^ the whole stomach was observed twice using primary white light imaging followed by second-generation narrow-band imaging in immediate succession (2258 participants) or primary second-generation narrow-band imaging followed by white light imaging in immediate succession (2265 participants). However, for each primary examination, 25% of the detected early GCs were missed, even though the examinations were conducted by expert endoscopists.^5^ This result suggested that detection of early GC remained difficult with the single observation method used in the current era, even with the advancement of endoscopic technologies. On the other hand, intensive endoscopic observation using both white light and second-generation narrow-band imaging in a single examination may be able to reduce the subsequent detection of early GC.^5^ The present case-control study was a secondary analysis of this randomized clinical trial,^5^ in which surveillance endoscopy was preplanned and scheduled between 9 and 15 months after index endoscopy to identify missed early GC. The study aimed to assess the rate of newly detected GC and risk factors associated with new GC detected within 15 months after intensive index endoscopy.

## Methods

### Study Design

This case-control study was a preplanned secondary analysis of a multicenter prospective randomized clinical trial that compared the detectability of early GC using white light imaging vs second-generation narrow-band imaging. The clinical trial was conducted at 13 hospitals in Japan between October 1, 2014, and September 22, 2017, with follow-up of 15 months. Data were analyzed from December 26, 2019, to April 21, 2021. The clinical trial protocol was approved by the review boards of all participating institutions and adhered to the Declaration of Helsinki.^6^ All participants provided written informed consent for the clinical trial and all secondary analyses. The current study followed the reporting guideline for case series studies proposed by Kempen.^7^

### Participants

We selected patients with a high risk of GC to maximize the detection rate of early GC.^5^ Several studies^8,9,10,11^ had reported that the incidence of GC development increased in patients with gastric neoplasm or esophageal cancer. Therefore, we included patients aged 20 to 85 years if they met 1 of the following criteria: (1) a history of endoscopic resection for an esophageal cancer or gastric neoplasm, (2) a current esophageal cancer or gastric neoplasm, or (3) a history of chemotherapy and/or radiotherapy for the treatment of esophageal cancer. The exclusion criteria were previously described.^5^

### Index Endoscopy

Patients were randomized on a 1:1 ratio to the white light imaging group or the second-generation narrow-band imaging group. Patients in the white light imaging group received index endoscopy consisting of nonmagnifying observations with white light imaging followed by second-generation narrow-band imaging, and patients in the second-generation narrow-band imaging group received second-generation narrow-band imaging followed by white light imaging. Therefore, the whole stomach was observed twice with primary and secondary examination performed in immediate succession. Index endoscopy was performed after systematic screening of the stomach, as proposed by Yao.^12^ Endoscopic images of the observation sites were recorded to the electromagnetic device during both white light and second-generation narrow-band imaging examinations. If target lesions suspected of being early GC were detected, they were immediately observed with near-focus narrow-band imaging. The diagnostic performance of near-focus narrow-band imaging for the diagnosis of early GC has been reported previously.^13^ After near-focus narrow-band imaging observation, biopsies were obtained from all target lesions. Endoscopists (including Y.Y., N.Y., T.Y., T.H., N.U., N.K., S.H., K.Y., S.A., C.K., C.Y., K.O., H.D., and M.M.) were required to be board-certified fellows of the Japan Gastroenterological Endoscopy Society or to have equivalent qualifications.

### Surveillance Endoscopy

We preplanned the performance of surveillance endoscopic examination to evaluate the rate of newly detected GC. Surveillance endoscopy was scheduled between 9 and 15 months after index endoscopy. If more than 1 surveillance endoscopy was conducted during that period, the earliest endoscopy was used for evaluation. If endoscopy was performed earlier than 9 months after index endoscopy only, that endoscopy was substituted for surveillance endoscopy. Surveillance endoscopy did not include a second review examination after endoscopic resection or emergency endoscopy. Unlike the index endoscopy, the surveillance endoscopy did not specify the type of endoscopic system used, the type of image-enhanced endoscopy used, the use of image-enhanced endoscopy, the use of chromoendoscopy, or the background and experience level of the endoscopists. However, board-certified fellows or endoscopists with equivalent qualifications performed most surveillance endoscopies for which image-enhanced endoscopy, chromoendoscopy, or magnifying observations were available.

### Definition of Newly Detected Gastric Cancer

Newly detected GC was defined as cancer detected within 15 months after index endoscopy. Lesions corresponding to target lesions detected during index endoscopy were excluded because biopsies had already been taken from all target lesions.^5^ Histological evaluation was based on the revised Vienna classification; tumors classified in category 4 (mucosal high-grade neoplasia) and category 5 (submucosal invasion by carcinoma) were diagnosed as GC, whereas lesions classified in categories 1 through 3 were diagnosed as noncancers.^14^

### Data Collection for Secondary Analysis

In this secondary analysis, some patients’ clinical and behavioral characteristics, such as an endoscopic finding of gastric mucosal atrophy, *Helicobacter pylori* infection status, aspirin use, statin use, smoking status, and alcohol consumption, were collected retrospectively. Gastric mucosal atrophy was categorized according to the Kimura–Takemoto classification system.^15^ In addition, for patients with newly detected GC, the archived endoscopic images obtained during index endoscopy were reviewed by endoscopists at all participating institutions to evaluate whether lesions were missed. Data were collected about the following: (1) whether archived image locations corresponding to newly detected GC were present or absent, (2) whether lesions in archived images corresponding to newly detected GC were detectable or not detectable, and (3) whether the retrospective diagnosis was presence of cancer or absence of cancer. A retrospective diagnosis of cancer was made if at least 1 of the following characteristics was found in index endoscopic images: (1) an area with an irregular margin, (2) an area with an irregular discoloration, or (3) an area with an irregular surface.

### Outcomes

The primary end point was the rate of new GC detected within 15 months after index endoscopy. The secondary end points were risk factors and clinical outcomes associated with new GC detected within 15 months after index endoscopy. Curability of endoscopic resection was histologically evaluated based on criteria from the Gastric Cancer Treatment Guidelines of the Japanese Gastric Cancer Association.^16,17^

### Statistical Analysis

The new GC detection rate was analyzed in the per protocol population, which was defined as all patients who received surveillance endoscopy within 15 months after index endoscopy. To clarify the risk factors associated with newly detected GC, a 1:1 matched control group was created involving patients in the study population who did not have new GC detected within 15 months of index endoscopy. Matching and selection of patients were conducted by an independent data center separate from the investigators. Factors used for matching were age, sex, participating institution, history of neoplasm at randomization, and period of registration. Categorical variables were expressed as frequencies with percentages, and continuous variables were expressed as means with SDs. Differences in proportions between the 2 groups were evaluated using a Fisher exact test. Multivariate analysis was performed using conditional logistic regression with partial likelihood. In the multivariate analysis, factors such as age and sex were excluded because they were used for matching; *H. pylori* infection status was also excluded because the control group had a nonnegligible amount of missing data for this variable. Missing unknown values were classified as *none*. For aspirin use, statin use, and alcohol consumption, unknown values were classified as *absent*. For smoking status, unknown values were classified as *never*. The significance threshold was 2-sided *P* = .05. All statistical analyses were performed using JMP software, version 14 (JMP Statistical Discovery LLC).

## Results

### Baseline Patient Characteristics

Among 4575 patients assessed for clinical trial eligibility between September 2014 and September 2017, 4523 patients (mean [SD] age, 70.6 [7.5] years; 3527 men [78.0%] and 996 women [22.0%]; all of Japanese ethnicity) were randomized, with 2258 patients receiving primary white light imaging followed by secondary second-generation narrow-band imaging (white light imaging group) and 2265 patients receiving primary second-generation narrow-band imaging followed by secondary white light imaging (second-generation narrow-band imaging group). Overall, 773 patients (17.1%) had a history of esophageal cancer, and 3750 patients (82.9%) had a history of gastric neoplasm. After randomization, 4472 patients received index endoscopy.

For the secondary analysis, 107 patients with newly detected GC were included in the case group, and 107 patients without newly detected GC were included in the matched control group. The case vs control groups were well balanced with regard to age (mean [SD], 71.7 [7.2] years vs 71.8 [7.0] years), sex (94 men [87.9%] and 13 women [12.1%] in each group), and history of gastric neoplasm (82 patients [76.6%] vs 87 patients [81.3%]) because these characteristics were used for matching. The number of patients with a history of esophageal cancer was also well balanced between the case group (18 patients [16.8%]) and the control group (19 patients [17.8%]). Additional characteristics of patients in the 2 groups are shown in Table 1.

**Table 1.** Patient Characteristics

Characteristic	Patients, No. (%)	
	Case group with newly detected GC	Matched control group without newly detected GC
Total patients, No.	107	107
Age, mean (SD), y	71.7 (7.2)	71.8 (7.0)
Sex		
Male	94 (87.9)	94 (87.9)
Female	13 (12.1)	13 (12.1)
History of esophageal cancer		
Absent	89 (83.2)	88 (82.2)
Present	18 (16.8)	19 (17.8)
History of gastric neoplasm^a^		
Absent	25 (23.4)	20 (18.7)
Present	82 (76.6)	87 (81.3)
Gastric mucosal atrophy^b^		
Closed type	11 (10.3)	33 (30.8)
Open type	96 (89.7)	74 (69.2)
*Helicobacter pylori* infection		
Positive	36 (33.6)	19 (17.8)
Positive and eradicated	67 (62.6)	48 (44.9)
Negative	3 (2.8)	9 (8.4)
Unknown	1 (0.9)	31 (29.0)
Aspirin use		
Absent	100 (93.5)	90 (84.1)
Present	6 (5.6)	13 (12.1)
Unknown	1 (0.9)	4 (3.7)
Statin use		
Absent	91 (85.0)	81 (75.7)
Present	15 (14.0)	19 (17.8)
Unknown	1 (0.9)	7 (6.5)
Smoking status		
Present	11 (10.3)	10 (9.3)
Former	57 (53.3)	57 (53.3)
Never	27 (25.2)	25 (23.4)
Unknown	12 (11.2)	15 (14.0)
Alcohol consumption		
Absent	35 (32.7)	36 (33.6)
Present	67 (62.6)	59 (55.1)
Unknown	5 (4.7)	12 (11.2)

Abbreviation: GC, gastric cancer.

^a^ Gastric neoplasms included GCs and gastric adenomas.

^b^ Presence of gastric mucosal atrophy was based on the classification system proposed by Kimura and Takemoto.^15^

### Detection Rate of Index vs Surveillance Endoscopy

During index endoscopy, early GCs were detected in 133 of 4472 patients, representing an early GC detection rate of 3.0%. The detection rates of early GC were 1.0% (8 of 773 patients) among those with a history of esophageal cancer and 3.3% (125 of 3750 patients) among those with a history of gastric neoplasm. Among 133 patients, we detected early GC in 97 patients (72.9%) on the first examination and 36 patients (27.1%) on the second examination.

**Figure 1.**
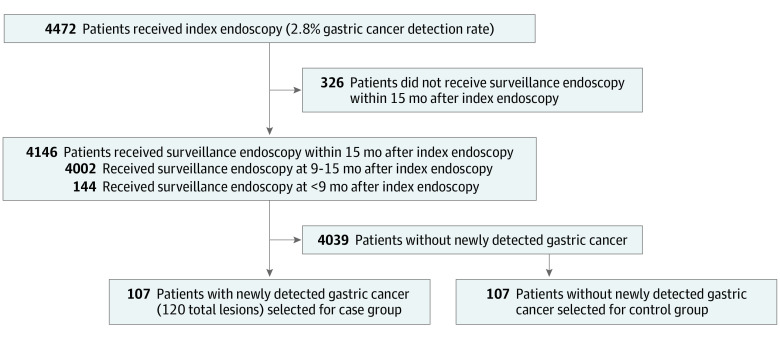
Flowchart of Study Patients

We performed surveillance endoscopy in 4146 of 4472 patients (92.7%) within 15 months after index endoscopy; of those, 4002 patients (96.5%) received preplanned surveillance endoscopy between 9 and 15 months after index endoscopy, and 144 patients (3.5%) received endoscopy earlier than 9 months only. We detected 120 new GCs in 107 of 4146 patients (2.6%) within 15 months after index endoscopy (Figure 1). Among 120 new GCs, 6 lesions were incidentally detected in the resected specimen of the main lesion; 3 of those lesions were in surgical specimens, and 3 were in endoscopic submucosal dissection specimens. With regard to timing of the detection of new GCs, 4 lesions were identified in the resected specimens of cancers detected by index endoscopy, and 2 lesions were identified in the resected specimens of cancers detected by surveillance endoscopy. Five of 6 lesions were smaller than 5 mm and were difficult to detect by endoscopy.

### Risk Factors

The 1:1 multivariate matched case-control analysis of patients with vs without newly detected GC revealed that open-type atrophic gastritis (odds ratio, 6.00; 95% CI, 2.25-16.01; *P* < .001) and early GC detection during index endoscopy (odds ratio, 4.67; 95% CI, 1.08-20.21; *P* = .04) were independent risk factors associated with new GC development (Table 2). Among 133 patients with GC detected by index endoscopy, 110 (82.7%) received surveillance endoscopy within 15 months after index endoscopy; the rate of newly detected GC was 10.9% (12 of 110 patients).

**Table 2.** Risk Factors Associated With Newly Detected Gastric Cancer

Factor	Patients, No. (%)		Univariate analysis, OR (95% CI)	*P* value	Multivariate analysis, OR (95% CI)	*P* value
	Case group (n = 107)	Control group (n = 107)				
History of esophageal cancer						
Absent	89 (83.2)	88 (82.2)	1 [Reference]	.79	1 [Reference]	.22
Present	18 (16.8)	19 (17.8)	0.88 (0.32-2.41)		0.47 (0.14-1.56)	
History of gastric neoplasm^a^						
Absent	25 (23.4)	20 (18.7)	1 [Reference]	.34	1 [Reference]	.32
Present	82 (76.6)	87 (81.3)	0.69 (0.32-1.48)		0.65 (0.28-1.51)	
Gastric mucosal atrophy^b^						
Closed type	11 (10.3)	33 (30.8)	1 [Reference]	<.001	1 [Reference]	<.001
Open type	96 (89.7)	74 (69.2)	4.14 (1.82-9.46)		6.00 (2.25-16.01)	
Aspirin use						
Absent	101 (94.4)	94 (87.9)	1 [Reference]	.12	1 [Reference]	.27
Present	6 (5.6)	13 (12.1)	0.46 (0.18-1.21)		0.52 (0.16-1.69)	
Statin use						
Absent	92 (86.0)	88 (82.2)	1 [Reference]	.47	1 [Reference]	.32
Present	15 (14.0)	19 (17.8)	0.77 (0.37-1.57)		0.65 (0.27-1.54)	
Smoking status						
Absent	96 (89.7)	97 (90.7)	1 [Reference]	.81	1 [Reference]	.92
Present	11 (10.3)	10 (9.3)	1.13 (0.43-2.92)		1.06 (0.34-3.30)	
Alcohol consumption						
Absent	40 (37.4)	48 (44.9)	1 [Reference]	.25	1 [Reference]	.20
Present	67 (62.6)	59 (55.1)	1.40 (0.79-2.49)		1.57 (0.79-3.11)	
No. of index endoscopies						
1	80 (74.8)	76 (71.0)	1 [Reference]	.42	1 [Reference]	.67
≥2	27 (25.2)	31 (29.0)	0.71 (0.32-1.61)		0.81 (0.31-2.11)	
Early GC detection during index endoscopy						
Absent	95 (88.8)	104 (97.2)	1 [Reference]	.03	1 [Reference]	.04
Present	12 (11.2)	3 (2.8)	4.00 (1.13-14.18)		4.67 (1.08-20.21)	

Abbreviations: GC, gastric cancer; OR, odds ratio.

^a^ Gastric neoplasms included gastric cancers and gastric adenomas.

^b^ Gastric mucosal atrophy was based on the classification proposed by Kimura and Takemoto.^15^

### Lesion Characteristics and Clinical Outcomes

The characteristics of the 120 new GCs detected within 15 months after index endoscopy are shown in eTable 1 in the Supplement. Most of the newly detected lesions (113 lesions [85.8%]) were smaller than 20 mm. With regard to morphological features, lesions were predominantly flat or depressed (97 lesions [80.8%]). The clinical outcomes of these 120 lesions are shown in the eFigure in the Supplement, and the clinicopathological characteristics of 111 resected newly detected GCs are shown in eTable 2 in the Supplement. Overall, 100 early GCs (90.1%) were endoscopically treated, with 93 curative resections. Eleven early GCs were treated with surgical procedures, and 7 GCs were within endoscopic curative resection criteria. In total, 100 newly detected GCs (90.1%) were within endoscopic curative resection criteria, and 11 detected GCs (9.9%) were not. With regard to depth of invasion, 97 lesions (87.4%) were mucosal cancer.

**Figure 2.**
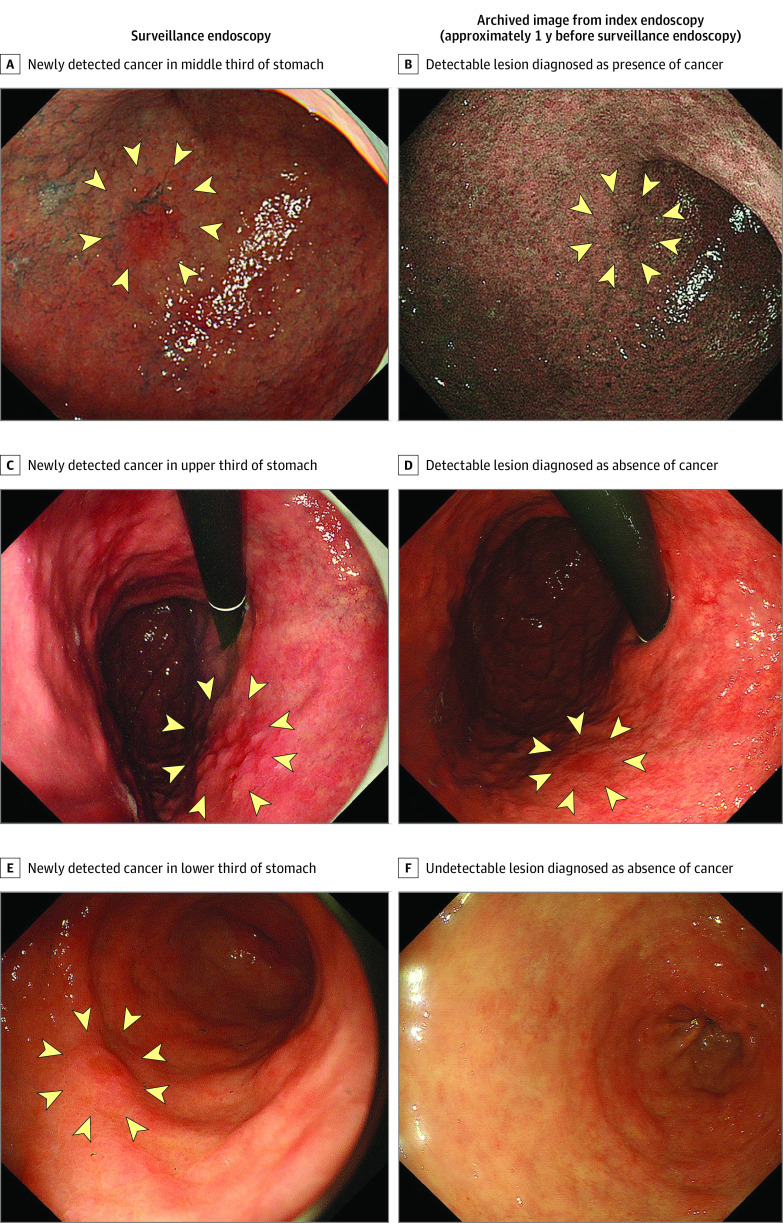
New Gastric Cancers Detected by Surveillance Endoscopy vs Archived Images From Index Endoscopy

Results from the review of archived endoscopic images obtained during index endoscopy (white light and narrow-band imaging) among patients with newly detected GCs are shown in Table 3. Among 120 new GCs, 60 lesions (50.0%) were not detected in the archived images, and 57 lesions (47.5%) were detected. A total of 21 lesions (17.5%) were retrospectively diagnosed as cancer, and 96 lesions (80.0%) were retrospectively diagnosed as noncancer. Representative images are shown in Figure 2. Three lesions could not be evaluated because the archived images with locations corresponding to newly detected GCs were absent.

**Table 3.** Review of Archived Images From Index Endoscopy and New Gastric Cancer Detected on Surveillance Endoscopy^a^

Archived image locations corresponding to newly detected gastric cancer	Lesions in archived images corresponding to newly detected gastric cancer	Retrospective diagnosis	Lesions, No. (%) (n = 120)
Present	Not detectable	Absence of cancer	60 (50.0)
Present	Detectable	Absence of cancer	36 (30.0)
Present	Detectable	Presence of cancer	21 (17.5)
Absent	Not evaluated	Not evaluated	3 (2.5)

^a^ Index endoscopy included both white light and narrow-band imaging.

## Discussion

This case-control study involving secondary analysis of a randomized clinical trial revealed that the rate of new GC detected within 15 months after intensive endoscopic examination was 2.6%, which was similar to that of index endoscopy (3.0%). The presence of open-type atrophic gastritis and the detection of GC during index endoscopy were independent risk factors associated with new GC development among patients with a high risk of GC and a history of gastric or esophageal cancer. Although board-certified endoscopists observed the whole stomach twice using white light and second-generation narrow-band imaging, index endoscopy was not associated with reductions in the detection rate of new GC after 1 year. These findings suggest that repeat endoscopy may be necessary for patients with a high risk of GC, especially those with atrophic gastritis or a history of GC.

To our knowledge, this study is the first large prospective analysis to clarify the detection rate of new GC within approximately 1 year after an intervention designed to help patients become free of GC using intensive index endoscopy. The performance of surveillance endoscopy was preplanned in the clinical trial protocol, achieving a follow-up rate of 92.7%. The results of this analysis may provide new reference criteria for the detection rate of new GC in patients with high risk.

Early detection of GC allows patients to receive minimally invasive treatment, potentially leading to better quality of life.^18,19^ In this study, 90.1% of newly detected GCs met the endoscopic curative resection criteria,^16^ although 9.9% did not meet criteria despite 1-year surveillance. It is possible that a prolonged surveillance interval may result in delay of GC detection and subsequent reduction of curability. For this reason, surveillance within approximately 1 year after detection of early GC is warranted for patients at high risk of developing GC.

Overall, 82.9% of patients enrolled in this study had a history of endoscopic resection of gastric neoplasm. Metachronous GC is generally defined as a newly developed cancer detected at least 1 year after endoscopic resection, and the annual incidence of metachronous GC has been reported to be 2.5% to 4.8%.^8,20,21,22,23^ In this study, the detection rate of new GC was consistent with the annual incidence of metachronous GC. However, GC detected within approximately 1 year after index endoscopy may have been cancers that were missed. Previous studies have reported the rate of missed gastric lesions as 0.6% to 11.6%,^24,25^ but the definition of missed lesions has varied.^26,27^ Although missed lesions were defined as cancers or neoplasms detected within 6 months to 3 years after endoscopic submucosal dissection or negative gastroscopic results, respectively,^24,28^ it is generally difficult to distinguish missed cancer from newly developed cancer. To determine whether we missed any early GCs, we reviewed the archived endoscopic images obtained during index endoscopy in patients with newly detected GC. We could not identify 60 lesions (50.0%) corresponding to newly detected GC in the archived images. It is possible that these lesions were newly developed cancers that became clinically visible in approximately 1 year. In contrast, we could identify 57 lesions (47.5%) corresponding to newly detected GC in the archived images. We could not eliminate the possibility that these lesions were missed, but only 21 lesions (17.5%) were retrospectively diagnosed as cancers. Our review of the archived images revealed that 80.0% of the newly detected GCs were retrospectively diagnosed as noncancers, even after an entire inspection of the stomach using 2 imaging modalities (white light and second-generation narrow band). These results suggest that new GC detected approximately 1 year after index endoscopy could be defined as metachronous rather than synchronous cancer.

In addition, widespread eradication of *H. pylori* may create difficulty in diagnosing early GC because of the increase in gastritis-like early GC (ie, gastritis-like appearance correlates with the superficial replacement of nonneoplastic epithelium of the cancer after eradication).^29,30,31^ The results of the current study suggest that limitations remain with regard to endoscopic detection of early GC despite the development of new endoscopic technologies. This study’s findings also revealed that open-type atrophic gastritis and detection of early GC during index endoscopy were independent risk factors associated with newly detected GC during surveillance endoscopy. Previous studies^22,30,32,33^ found that older age, multiple initial early GCs, severe gastric mucosal atrophy, and persistent *H. pylori* infection were risk factors for metachronous GC. In contrast, our study found that detection of GC within 1 year after index endoscopy was a risk factor associated with detection of new GC during surveillance endoscopy.

Endoscopic surveillance intervals based on individual patient risk are recommended in several guidelines. According to MAPSII (Management of Epithelial Precancerous Conditions and Lesions in the Stomach) guidelines^34^ and 2020 guidelines from the American Gastroenterological Association Institute,^35^ patients with advanced stages of atrophic gastritis should be followed up with a high-quality endoscopic examination every 3 years. The results of the current study suggest that shorter surveillance intervals may be preferable for patients with advanced stages of atrophic gastritis. However, it is difficult to apply the results of this study equally to populations with high and low GC incidence. In populations with low GC incidence, it would be important to determine surveillance intervals based on individual risk factors, such as the presence of atrophic gastritis and a recent history of early GC.

### Limitations

This study has several limitations. First, surveillance endoscopy did not specify the type of image-enhanced endoscopy used or the background or experience level of the endoscopists. Therefore, we could not identify the precise proportion of image-enhanced endoscopy used or the experience level of endoscopists. Second, some patient data were collected retrospectively. Thus, the control group includes patients with unknown *H. pylori* infection status. Third, we reviewed the archived images with foreknowledge that certain lesions were cancer. Therefore, the 17.5% of lesions that were diagnosed as cancer might represent a high estimate. Fourth, the sample size for a 1:1 matched case-control analysis is small.

## Conclusions

In this case-control study involving secondary analysis of a randomized clinical trial, the use of intensive index endoscopy in patients at high risk of GC was not associated with reductions in the detection rate of new GCs detected after 1 year, although the whole stomach was observed twice with white light and second-generation narrow-band imaging. New GC detection was associated with the presence of open-type atrophic gastritis and the detection of early GC by index endoscopy. These results suggest that, to detect new GC at an early stage, 1-year surveillance after detection of early GC in patients with high risk is warranted.

## Notes

Supplement. **eTable 1.** Lesion Characteristics of Newly Detected Gastric Cancer **eTable 2.** Clinicopathological Characteristics of Resected Newly Detected Gastric Cancer **eFigure.** Outcomes of 120 New Gastric Cancer Lesions
