# Supplementary material for: Assessment of Outcomes From 1-Year Surveillance After Detection of Early Gastric Cancer Among Patients at High Risk in Japan
Source: JAMA Netw Open. 2022 Aug 19;5(8):e2227667. doi: 10.1001/jamanetworkopen.2022.27667 (PMC9391963; doi:10.1001/jamanetworkopen.2022.27667)
Supplement: Supplement. — eTable 1. Lesion Characteristics of Newly Detected Gastric Cancer eTable 2. Clinicopathological Characteristics of Resected Newly Detected Gastric Cancer eFigure. Outcomes of 120 New Gastric Cancer Lesions [file jamanetwopen-e2227667-s001.pdf]

Supplementary Online Content

Yamamoto Y, Yoshida N, Yano T, et al. Assessment of outcomes from 1-year surveillance after detection of early gastric cancer among patients with high risk in Japan. *JAMA Netw Open*. 2022;5(8):e2227667. doi:10.1001/jamanetworkopen.2022.27667

eTable 1. Lesion Characteristics of Newly Detected Gastric Cancer

eTable 2. Clinicopathological Characteristics of Resected Newly Detected Gastric Cancer

eFigure. Outcomes of 120 New Gastric Cancer Lesions

This supplementary material has been provided by the authors to give readers additional information about their work.

© 2022 Yamamoto Y et al. *JAMA Network Open*.

eTable 1. Lesion Characteristics of Newly Detected Gastric Cancer (n = 120)

Lesion characteristics	n (%)
Detection interval after index Endoscopy	
within 8 months	32 (26.7)
9-15 months	88 (73.3)
Location [a]	
Upper	31 (25.8)
Anterior	8 (6.7)
Lesser curvature	9 (7.5)
Posterior	7 (5.8)
Greater curvature	7 (5.8)
Middle	46 (38.3)
Anterior	12 (10.0)
Lesser curvature	21 (17.5)
Posterior	6 (5.0)
Greater curvature	7 (5.8)
Lower	43 (35.8)
Anterior	9 (7.5)
Lesser curvature	9 (7.5)
Posterior	6 (5.0)
Greater curvature	19 (15.8)
Size (endoscopic findings)	
10mm≦	68 (56.7)
10-20mm	35 (29.1)
20mm＞	17 (14.2)
Color	
Redness	74 (61.7)
No change in color	26 (21.7)
Discolored	17 (14.2)
Unknown	3 (2.5)
Macroscopic type [a]	
Elevated	18 (15.0)
Flat/Depressed	97 (80.8)
Unknown	5 (4.2)

[a]Tumor location and macroscopic type were classified according to the Japanese Classification of Gastric Carcinoma.[33]

© 2022 Yamamoto Y et al. *JAMA Network Open*.

eTable 2. Clinicopathological Characteristics of Resected Newly Detected Gastric Cancer (n = 111)

Clinicopathologic characteristics	n (%)
Resection method	
Endoscopic resection	100 (90.1)
Surgery	11 (9.9)
Size (pathologic findings)	
10mm≦	59 (53.1)
10-20mm	36 (32.4)
20mm＞	16 (14.4)
Histology [a]	
Differentiated	96 (86.5)
Undifferentiated	12 (10.8)
Others	3 (2.7)
Depth of invasion [a]	
pT1a	97 (87.3)
pT1b1	6 (5.4)
pT1b2	6 (5.4)
pT2 or deeper	2 (1.8)
Lymphatic invasion	
Negative	106 (95.5)
Positive	5 (4.5)
Vascular invasion	
Negative	107 (96.4)
Positive	4 (3.6)
Endoscopic curative resection criteria [b]	
Within criteria	100 (90.1)
Beyond criteria	11 (9.9)

[a]Histologic classification and depth of invasion were classified according to the Japanese Classification of Gastric Carcinoma.[33]

[b]Endoscopic curative resection was classified according to the Gastric Cancer Treatment Guidelines by Japanese Gastric Cancer Association.

© 2022 Yamamoto Y et al. *JAMA Network Open*.

eFigure. Outcomes of 120 New Gastric Cancer Lesions

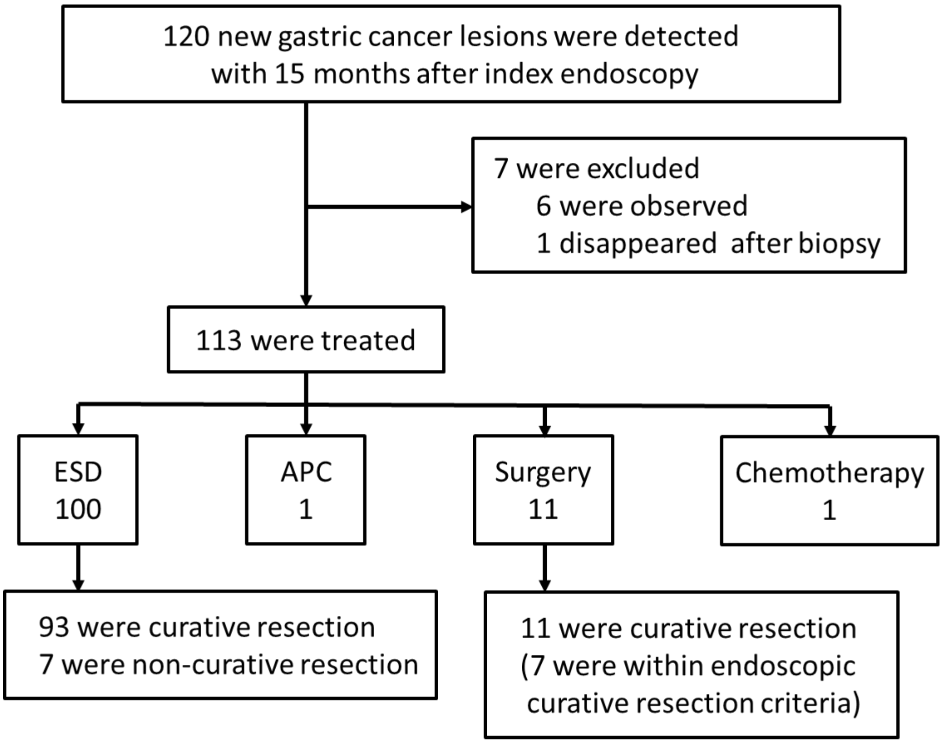

ESD: endoscopic submucosal dissection; APC: argon plasma coagulation

© 2022 Yamamoto Y et al. *JAMA Network Open*.
